# Association between smoking status and subclinical coronary atherosclerosis in asymptomatic Korean individuals

**DOI:** 10.4178/epih.e2024064

**Published:** 2024-07-16

**Authors:** Hyeji Lee, Jinhee Ha, Kyung Sun Park, Young-Jee Jeon, Sangwoo Park, Soe Hee Ann, Yong-Giun Kim, Yongjik Lee, Woon Jung Kwon, Seong Hoon Choi, Seungbong Han, Gyung-Min Park

**Affiliations:** 1 Department of Emergency Medicine, Ulsan University Hospital, University of Ulsan College of Medicine, Ulsan, Korea; 2 Department of Dentistry, Ulsan University Hospital, University of Ulsan College of Medicine, Ulsan, Korea; 3 Department of Nephrology, Ulsan University Hospital, University of Ulsan College of Medicine, Ulsan, Korea; 4 Department of Family Medicine, Ulsan University Hospital, University of Ulsan College of Medicine, Ulsan, Korea; 5 Department of Cardiology, Ulsan University Hospital, University of Ulsan College of Medicine, Ulsan, Korea; 6 Department of Thoracic and Cardiovascular Surgery, Ulsan University Hospital, University of Ulsan College of Medicine, Ulsan, Korea; 7 Department of Radiology, Ulsan University Hospital, University of Ulsan College of Medicine, Ulsan, Korea; 8 Department of Biostatistics, Korea University College of Medicine, Seoul, Korea; 9 Basic-Clinic Convergence Research Institute, University of Ulsan, Ulsan, Korea

**Keywords:** Smoking, Smokers, Ex-smokers, Non-smokers, Coronary artery disease, Atherosclerosis

## Abstract

**OBJECTIVES:**

In this study, we sought to evaluate the association between smoking status and subclinical coronary atherosclerosis, as detected by coronary computed tomography angiography (CCTA), in asymptomatic individuals.

**METHODS:**

We retrospectively analyzed 9,285 asymptomatic participants (mean age, 53.7±8.0 years; n=6,017, 64.8% male) with no history of coronary artery disease (CAD) who had undergone self-referred CCTA. Of these participants, 4,333 (46.7%) were considered never smokers, 2,885 (31.1%) former smokers, and 2,067 (22.3%) current smokers. We assessed the degree and characteristics of subclinical coronary atherosclerosis using CCTA, with obstructive CAD defined as a diameter stenosis of at least 50%.

**RESULTS:**

Compared with never-smokers, former smokers exhibited no significant differences in the probabilities of obstructive CAD, any coronary plaque, calcified plaque, or mixed plaque, as determined using adjusted odds ratios (aORs; p>0.05 for all). However, the risk of non-calcified plaque was significantly higher in former smokers (aOR, 1.34; 95% confidence interval [CI], 1.00 to 1.78; p=0.048). Current smokers had significantly higher rates of obstructive CAD (aOR, 1.46; 95% CI, 1.10 to 1.96; p=0.010), any coronary plaque (aOR, 1.41; 95% CI, 1.20 to 1.65; p<0.001), calcified plaque (aOR, 1.32; 95% CI, 1.13 to 1.55; p=0.001), non-calcified plaque (aOR, 1.72; 95% CI, 1.28 to 2.32; p<0.001), and mixed plaque (aOR, 2.00; 95% CI, 1.39 to 2.86; p<0.001) compared to never smokers.

**CONCLUSIONS:**

This cross-sectional study revealed a significant association between current smoking and subclinical coronary atherosclerosis, as detected on CCTA. Additionally, former smoking demonstrated an association with non-calcified plaque, indicating elevated cardiovascular risk.

## GRAPHICAL ABSTRACT

[Fig f2-epih-46-e2024064]

## Key Message

• Current smoking is an independent predictor of subclinical coronary atherosclerosis.

• Former smoking is associated with non-calcified plaque reflecting the early atherosclerosis and vulnerability.

• Appropriate strategies for smoking cessation are needed to prevent subclinical coronary atherosclerosis.

## INTRODUCTION

Coronary artery disease (CAD) is the leading cause of death worldwide [1]. Previous epidemiological studies have identified smoking as a major risk factor for CAD [2-4]. Smoking not only independently increases the risk for CAD but also exerts a multiplicative effect on CAD risk when combined with other traditional risk factors [5]. However, limited data are available regarding the influence of smoking status on the risk of subclinical coronary atherosclerosis in asymptomatic individuals. With the advent of multidetector computed tomography, coronary computed tomography angiography (CCTA) enables comprehensive assessment of CAD, including lesion location, disease severity, and plaque characteristics [6]. Although previous studies using CCTA have investigated the impact of smoking status on the risk of CAD, they did not focus on asymptomatic participants, and the results were inconsistent with earlier findings [7,8]. Therefore, we aimed to evaluate the association between former or current smoking and subclinical coronary atherosclerosis in a large cohort of asymptomatic Korean participants who underwent self-referred CCTA for early detection of CAD.

## MATERIALS AND METHODS

### Study population

**Figure 1.**
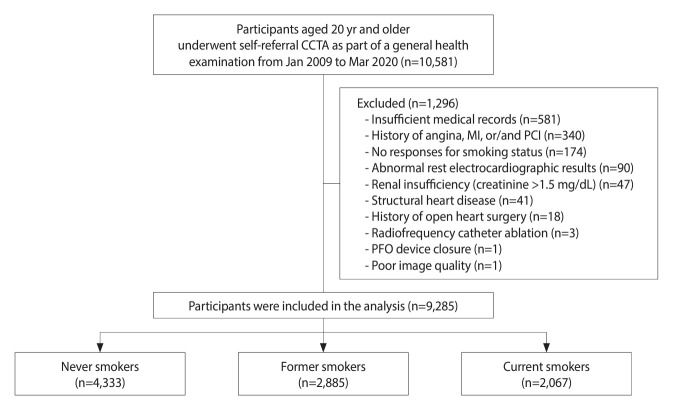
Overview of the study population. CCTA, coronary computed tomographic angiography; MI, myocardial infarction; PCI, percutaneous coronary intervention; PFO, patent foramen ovale.

Between January 2009 and March 2020, a total of 97,835 consecutive Korean individuals aged 19 years and older received a general health examination at the Health Promotion Center of Ulsan University Hospital. Among them, 10,581 participants who underwent self-referred CCTA were included in this study (Supplementary Material 1). Figure 1 details the participant selection process. Following the exclusion of ineligible patients, the final analysis comprised 9,285 individuals, as previously described [9].

### Clinical and laboratory measurements

We accessed clinical and laboratory data from the electronic medical records and clinical data warehouse platform of Ulsan University Hospital. During the general medical check-up, height, body weight, waist circumference, and blood pressure were measured according to standard procedures, as detailed in previous studies [9,10]. Levels of glucose, hemoglobin A1c, total cholesterol, low-density lipoprotein cholesterol, high-density lipoprotein cholesterol, triglycerides, creatinine, and C-reactive protein were measured. Left ventricular ejection fraction was determined using transthoracic echocardiography [9,10].

The study participants were categorized by smoking status as never, former, or current smokers. This classification was determined based on self-reported cigarette consumption. Individuals who had smoked fewer than 100 cigarettes in their lifetime were considered never-smokers. In contrast, those who had smoked 100 or more cigarettes were identified as either former or current smokers, contingent upon their present smoking practices [11]. To quantify smoking exposure, pack-years were used, calculated as the average number of cigarette packs smoked daily multiplied by the duration of smoking in years.

Obesity was defined as having a body mass index of 25 kg/m^2^ or higher, in line with the Asia-specific cut-off recommended by the World Health Organization. Diabetes mellitus was characterized by a fasting plasma glucose level of 126 mg/dL or above, a hemoglobin A1c level of 6.5% or higher, a self-reported history of diabetes, or current treatment for diabetes (either dietary or pharmacological) as indicated on the structured questionnaire. Hypertension was defined as a blood pressure of 140/90 mmHg or higher, a self-reported history of hypertension, or the use of antihypertensive medication. Hyperlipidemia was identified by a total cholesterol level of 240 mg/dL or higher, a self-reported history of hyperlipidemia, or the use of lipid-lowering medication. Family history of CAD was determined based on the presence of a first-degree relative of any age with CAD, as reported on the self-report questionnaire [12]. The 10-year CAD risk score was calculated based on previous guidelines [3].

### Coronary computed tomography angiography image acquisition and analysis

CCTA was performed using either a single-source, 256-slice computed tomography scanner (Brilliance iCT; Philips Healthcare, Best, The Netherlands) or a dual-source scanner (Somatom Definition Flash; Siemens, Erlangen, Germany). The CCTA protocol has been detailed previously [9,10]. CCTA images were interpreted by experienced cardiovascular radiologists and a cardiologist (WJK, SHC, and GMP, each with over 10 years of experience) using a dedicated workstation (Syngo.via, Siemens or Aquarius iNtuition, TeraRecon Inc., Durham, NC, USA). The coronary artery calcium score (CACS) was measured and categorized based on the Agatston score as 0, 1-10, 11-100, 101-400, or > 400 [13]. Plaques with more than 50% calcified tissue (density > 130 Hounsfield units) were classified as calcified, those with less than 50% calcium were categorized as mixed, and those without calcium were deemed non-calcified [14]. Diameter stenosis of 50% or greater was defined as obstructive CAD.

### Statistical analysis

Categorical variables are presented as frequencies with percentages, while continuous variables are expressed as means with standard deviations. We compared baseline patient characteristics across the 3 smoking status groups using one-way analysis of variance for continuous variables and the Pearson chi-square test for categorical variables. To explore the relationship between smoking status and subclinical coronary atherosclerosis as detected on CCTA, we employed both univariate and multivariable logistic regression models. Drawing on findings from previous epidemiological studies [2-4], we included clinically relevant variables such as age, sex, diabetes mellitus, hypertension, hyperlipidemia, obesity, family history of CAD, and C-reactive protein level as candidate adjustment variables in the multivariable logistic model. In this model, all variables except age were treated as categorical. In a further analysis, we divided participants into 5 groups, stratifying former and current smokers into 2 subgroups each based on median pack-years. These median values were determined to be 15 pack-years for former smokers and 20 for current smokers. Accordingly, former smokers with fewer than 15 pack-years were categorized as light former smokers, and those with 15 or more pack-years were considered heavy former smokers. Current smokers were similarly classified: those with fewer than 20 pack-years as light current smokers and those with 20 or more pack-years as heavy current smokers. All reported p-values are 2-sided, with values less than 0.05 considered to indicate statistical significance. Data manipulation and statistical analysis were performed using SPSS version 24 (IBM Corp., Armonk, NY, USA) and R version 4.0.2 (R Foundation for Statistical Computing, Vienna, Austria).

### Ethics statement

This retrospective cross-sectional study received approval from the Institutional Review Board of Ulsan University Hospital, Ulsan, Korea (IRB No. UUH 2020-12-033).

## RESULTS

### Characteristics of the study population

The health examination recipients who underwent CCTA were older and had a greater number of comorbid conditions compared to those who did not undergo CCTA (Supplementary Material 2). The mean age of study participants undergoing CCTA was 53.7±8.0 years, with 6,017 (64.8%) of participants being male. Within the study sample, 4,333 (46.7%) were categorized as never smokers, 2,885 (31.1%) as former smokers, and 2,067 (22.3%) as current smokers. Table 1 presents the baseline characteristics of the study population stratified by smoking status. Current smokers were younger and exhibited higher 10-year atherosclerotic cardiovascular disease risk scores compared to never and former smokers.

### Coronary computed tomography angiography findings

Table 2 displays the CCTA findings categorized by smoking status. The mean CACS among the study participants was 39.5±159.9. The average CACS was highest for former smokers, followed by current smokers and then never smokers (p<0.001). Coronary plaques of any type were detected in 3,121 participants (33.6%), while calcified, non-calcified, and mixed plaques were found in 2,884 (31.1%), 543 (5.8%), and 346 (3.7%) individuals, respectively. Coronary plaques of any type and calcified plaques were more prevalent among former smokers, whereas non-calcified and mixed plaques were more commonly observed in current smokers. Of the participants, 572 (6.2%) exhibited obstructive CAD (≥ 50% diameter stenosis) in at least 1 coronary artery as determined by CCTA. Obstructive CAD in the left main, left anterior descending, left circumflex, and right coronary arteries was found in 10 (0.1%), 433 (4.7%), 140 (1.5%), and 211 (2.3%) participants, respectively. Former smokers displayed a higher prevalence of obstructive CAD in at least 1 coronary artery compared to never-smokers and current smokers (p<0.001).

### Association between smoking status and subclinical coronary atherosclerosis

The relationship between smoking status and subclinical coronary atherosclerosis is detailed in Table 3. After adjusting for cardiovascular risk factors—including age, sex, diabetes mellitus, hypertension, hyperlipidemia, obesity, family history of CAD, and C-reactive protein level—no significant differences were observed in the adjusted odds ratios (aORs) for the presence of any coronary plaque, calcified plaque, mixed plaque, or obstructive CAD between former smokers and never smokers. However, the probability of exhibiting non-calcified plaque was significantly greater among former smokers (aOR, 1.34; 95% confidence interval [CI], 1.00 to 1.78; p=0.048).

In univariable analyses, a significant association was observed between current smoking and the presence of any subclinical coronary atherosclerosis (p<0.05 for all). Multivariable analyses further indicated that relative to never smokers, current smokers displayed significantly higher odds of obstructive CAD (aOR, 1.46; 95% CI, 1.10 to 1.96; p=0.010), any coronary plaque (aOR, 1.41; 95% CI, 1.20 to 1.65; p<0.001), calcified plaque (aOR, 1.32; 95% CI, 1.13 to 1.55; p=0.001), non-calcified plaque (aOR, 1.72; 95% CI, 1.28 to 2.32; p<0.001), and mixed plaque (aOR, 2.00; 95% CI, 1.39 to 2.86; p<0.001).

After excluding participants with no recorded smoking quantity (n=375), we categorized the remaining participants into 5 groups based on smoking amount. The distribution was as follows: 4,333 (48.6%) were never smokers, 1,291 (14.5%) were light former smokers, 1,383 (15.5%) were heavy former smokers, 848 (9.5%) were light current smokers, and 1,055 (11.8%) were heavy current smokers. We then examined the relationship of subclinical coronary atherosclerosis with smoking status and amount (Tables 4 and 5). Heavy current smoking displayed significant associations with all forms of coronary atherosclerosis, including coronary artery calcification, all 3 types of coronary plaque (calcified, non-calcified, and mixed), and obstructive CAD. Additionally, light current smoking and heavy former smoking were significantly associated with the presence of any coronary plaque (aOR 1.24; 95% CI, 1.02 to 1.52; p=0.033) and non-calcified plaque (aOR 1.57; 95% CI, 1.15 to 2.14; p=0.005), respectively (Table 5).

## DISCUSSION

The principal findings of this study are as follows: (1) current smoking emerged as an independent predictor of subclinical coronary atherosclerosis on CCTA after adjustment for cardiovascular risk factors; (2) former smoking was significantly associated with the presence of non-calcified plaques, but not calcified or mixed plaques; and (3) smoking amount was also associated with subclinical coronary atherosclerosis as detected on CCTA.

The relationship between smoking status and subclinical atherosclerosis has been explored in prior research using measures such as carotid intima-media thickness, ankle-brachial index, and coronary artery calcification [15-20]. Initial studies employing electron beam-computed tomography revealed an association between smoking status and coronary artery calcification [15,16,18,19]. Advances in computed tomography technology have enabled CCTA to provide extensive insights into coronary atherosclerosis, including lesion location, disease severity, and plaque characteristics. These characteristics encompass (1) the extent of coronary artery calcification (CACS); (2) the type of coronary plaque (calcified, non-calcified, or mixed); and (3) the presence of obstructive CAD [6]. However, a previous CCTA study yielded conflicting findings, indicating that calcified plaque and high CACS (> 100) did not correlate with smoking status [8]. Consequently, the link between smoking status and subclinical coronary atherosclerosis remains unclear. The present study aimed to evaluate this association by analyzing data from a large CCTA registry, following a careful exclusion process.

This study revealed that former smoking was significantly associated with non-calcified plaques, but not with calcified or mixed plaques. Non-calcified plaque contains fibrous and lipid tissue and represents an earlier stage of coronary atherosclerosis, while coronary plaque calcification is a later manifestation of the disease [21,22]. Additionally, the CACS indicates the stage of plaque progression and maturity [21,22], but it does not accurately reflect the true burden of atherosclerosis or the severity of coronary artery stenoses, particularly in younger individuals [23,24]. Research indicates that vulnerable plaques are predominantly non-calcified and non-stenotic lesions [25]. In diverse populations, the presence of non-calcified plaques on CCTA has been linked to adverse CAD events [26-28]. A prospective follow-up study employing CCTA showed that non-calcified plaques were associated with the culprit lesions in patients who later experienced acute coronary syndrome [26]. Moreover, non-calcified plaques were linked to cardiac events even in asymptomatic individuals [27]. A systematic meta-analysis also revealed that non-calcified plaques detected by CCTA could progress and confer an increased risk of acute coronary syndrome events [29]. The rapid decline in myocardial infarction risk observed after smoking cessation, as noted in some case-control studies, suggests that smoking may trigger the development of myocardial infarction [30]. In our study, the link between former smoking and non-calcified plaque suggests that smoking may play a role in early coronary atherosclerosis and plaque vulnerability. Accordingly, this study offers insights into the mechanisms by which smoking affects coronary atherosclerosis.

In contrast to former smoking, current smoking was consistently identified as an independent risk factor for any coronary plaque; calcified, non-calcified, and mixed plaques; and obstructive CAD, after adjusting for cardiovascular risk factors. The primary mechanisms driving smoking-induced atherogenesis include endothelial dysfunction and damage, increased levels and oxidation of proatherogenic lipids, induction of inflammation, and a shift toward a procoagulant state in the circulation [31]. Continuous and chronic exposure to smoking is likely linked to these mechanisms, contributing to the development of subclinical coronary atherosclerosis in current smokers. The international CONFIRM (Coronary CT Angiography Evaluation for Clinical Outcomes: An International Multicenter) registry has documented an elevated burden of atherosclerosis and a higher rate of all-cause mortality or non-fatal myocardial infarction in current smokers compared to never-smokers [7]. In our study, current smoking was also significantly associated with obstructive CAD, which is known to correlate with comparatively poor prognosis even in asymptomatic individuals [32,33]. Thus, our findings further underscore the importance of smoking cessation in the prevention of subclinical coronary atherosclerosis.

Furthermore, in the present study, the amount of smoking was associated with subclinical coronary atherosclerosis. Based on these and previous findings, smoking appears to play a key role not only in the initiation of coronary atherosclerosis but also in significantly contributing to the progression of CAD and related events. Prior cohort studies involving patients who underwent coronary artery calcium screening have identified a significant association between smoking and CACS, with smoking significantly increasing the risk of events in asymptomatic individuals who have a comparable calcium burden [15,18,19]. Moreover, smoking cessation has been shown to mitigate the development of coronary artery calcification [16]. This pattern has also been observed regarding the carotid intima-media thickness and ankle-brachial index, which are indicators of more advanced atherosclerosis [34]. Additionally, population-based studies have confirmed that smoking is a modifiable risk factor and that cessation can reduce cardiovascular events and mortality [21,35,36]. Consequently, smoking cessation is imperative to reduce the risk of subclinical coronary atherosclerosis and subsequent cardiac events.

Our study had several limitations. First, it was conducted at a single center. Additionally, since all participants chose to attend the hospital for a general health examination, selection bias may have impacted the results. Furthermore, as most of the former and current smokers were male, we lacked sufficient data to assess the association between smoking status and subclinical coronary atherosclerosis in female. Second, this study was a cross-sectional analysis without clinical follow-up data, which restricted our ability to evaluate the impact of smoking status on CAD events. Third, the smoking histories were obtained from a standardized self-report questionnaire completed before a general health examination provided by Korea’s National Health Insurance. Consequently, the data did not include certain details; for example, we lacked information on the age at which former smokers had quit. Fourth, our study population was exclusively Korean, potentially limiting the generalizability of our findings to other ethnic groups. Finally, CCTA has potential drawbacks, including radiation exposure, the need for contrast, and higher costs. Our study enrolled only volunteers, and the justification for using CCTA in asymptomatic individuals remains to be established.

In conclusion, this large observational study involving asymptomatic individuals who underwent CCTA demonstrated a significant association between current smoking and subclinical coronary atherosclerosis. Individuals who had never smoked displayed no such association. Additionally, former smoking displayed an association with non-calcified plaque, indicating a heightened risk of cardiovascular disease. Further studies are required to validate these findings.

## Figures and Tables

**Figure f2-epih-46-e2024064:**
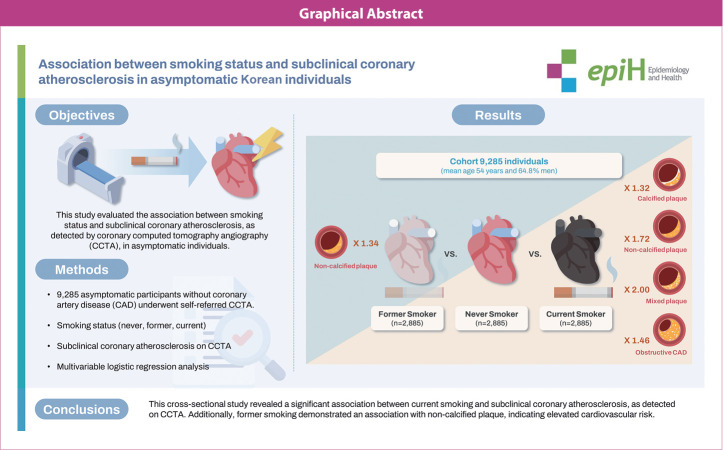


**Table 1.** Baseline characteristics of study participants according to smoking status

Characteristics	Smoking status			p-value
	Never smoker	Former smoker	Current smoker	
No. of patients	4,333 (46.7)	2,885 (31.1)	2,067 (22.3)	
Demographics				
Age (yr)	54.4±8.3	54.5±7.4	51.3±7.8	<0.001
Male	1,200 (27.7)	2,828 (98.0)	1,989 (96.2)	<0.001
Clinical characteristics or coexisting conditions				
Pack-years of smoking	-	16.7±13.7	22.6±14.8	<0.001
Body mass index (kg/m^2^)	23.7±3.0	24.7±2.7	24.5±2.9	<0.001
Waist circumference (cm)	83.7±7.8	87.4±7.2	87.1±7.5	<0.001
Systolic blood pressure (mmHg)	123.6±14.7	126.8±12.6	124.5±13.1	<0.001
Diastolic blood pressure (mmHg)	77.2±9.7	80.1±8.7	78.8±9.2	<0.001
Diabetes mellitus	458 (10.7)	393 (13.8)	348 (17.3)	<0.001
Hypertension	1,361 (31.7)	1,124 (39.4)	620 (30.7)	<0.001
Hyperlipidemia	742 (17.4)	540 (19.0)	373 (18.5)	0.195
Obesity^1^	1,311 (30.4)	1,201 (41.8)	863 (41.9)	<0.001
Previous stroke	23 (0.7)	12 (0.6)	6 (0.4)	0.511
Family history of CAD^2^	387 (9.1)	249 (8.8)	185 (9.2)	0.864
Fasting blood glucose (mg/dL)	93.5±19.7	98.7±22.7	99.4±27.1	<0.001
Glycated hemoglobin (%)	5.6±0.7	5.7±0.8	5.8±0.9	<0.001
Total cholesterol (mg/dL)	192.0±36.6	189.7±36.1	192.5±37.8	0.010
LDL cholesterol (mg/dL)	127.1±34.1	127.0±33.4	128.4±34.9	0.320
HDL cholesterol (mg/dL)	56.5±15.4	50.8±13.5	47.7±13.4	<0.001
Triglycerides (mg/dL)	99.8±60.8	122.3±80.5	144.2±94.1	<0.001
Creatinine (mg/dL)	0.8±0.2	0.9±0.2	0.9±0.2	<0.001
C-reactive protein ≥2 mg/L	18 (0.4)	17 (0.6)	17 (0.8)	0.122
Ejection fraction (%)	64.5±4.7	64.2±4.5	64.0±4.7	0.007
ASCVD risk score	4.4±6.0	7.2±6.3	10.6±7.4	<0.001

Values are presented as mean±standard deviation or number (%). CAD, coronary artery disease; LDL, low-density lipoprotein; HDL, high-density lipoprotein; ASCVD, atherosclerotic cardiovascular disease.

1 Defined as a body mass index ≥25 kg/m^2^.

2 Defined as CAD in a first-degree relative of any age.

**Table 2.** Coronary computed tomographic angiographic findings according to smoking status

Variables	Smoking status			p-value
	Never smoker	Former smoker	Current smoker	
Coronary artery calcium score				
Mean±SD	24.6±118.9	57.2±201.7	46.1±165.7	<0.001
0	3,316 (76.9)	1,688 (58.8)	1,289 (62.7)	<0.001
1-10	314 (7.3)	298 (10.4)	194 (9.4)	
11-100	435 (10.1)	534 (18.6)	334 (16.2)	
101-400	181 (4.2)	247 (8.6)	189 (9.2)	
>400	66 (1.5)	105 (3.7)	51 (2.5)	
Any coronary plaque	1,067 (24.6)	1,237 (42.9)	817 (39.5)	<0.001
Plaque characteristics				
Calcified	976 (22.5)	1,167 (40.5)	741 (35.8)	<0.001
Non-calcified	163 (3.8)	217 (7.5)	163 (7.9)	<0.001
Mixed	88 (2.0)	134 (4.6)	124 (6.0)	<0.001
Obstructive coronary artery disease	166 (3.8)	246 (8.5)	160 (7.7)	<0.001

Values are presented as number (%). SD, standard deviation.

**Table 3.** Association of smoking status with coronary computed tomographic angiographic findings

Variables^1^	Univariable	p-value	Multivariable^2^	p-value
Any coronary plaque				
Never smokers (1,067/4,333, 24.6%)	1.00 (reference)	-	1.00 (reference)	-
Former smokers (1,237/2,885, 42.9%)	2.30 (2.08, 2.54)	<0.001	1.13 (0.97, 1.31)	0.117
Current smokers (817/2,067, 39.5%)	2.00 (1.79, 2.24)	<0.001	1.41 (1.20, 1.65)	<0.001
Calcified plaque				
Never smokers (976/4,333, 22.5%)	1.00 (reference)	-	1.00 (reference)	-
Former smokers (1,167/2,885, 40.5%)	2.34 (2.11, 2.59)	<0.001	1.12 (0.97, 1.30)	0.131
Current smokers (741/2,067, 35.8%)	1.92 (1.71, 2.16)	<0.001	1.32 (1.13, 1.55)	0.001
Non-calcified plaque				
Never smokers (163/4,333, 3.8%)	1.00 (reference)	-	1.00 (reference)	-
Former smokers (217/2,885, 7.5%)	2.08 (1.69, 2.56)	<0.001	1.34 (1.00, 1.78)	0.048
Current smokers (163/2,067, 7.9%)	2.19 (1.75, 2.74)	<0.001	1.72 (1.28, 2.32)	<0.001
Mixed plaque				
Never smokers (88/4,333, 2.0%)	1.00 (reference)	-	1.00 (reference)	-
Former smokers (134/2,885, 4.6%)	2.35 (1.79, 3.09)	<0.001	1.14 (0.80, 1.62)	0.460
Current smokers (124/2,067, 6.0%)	3.08 (2.33, 4.07)	<0.001	2.00 (1.39, 2.86)	<0.001
Obstructive coronary artery disease				
Never smokers (166/4,333, 3.8%)	1.00 (reference)	-	1.00 (reference)	-
Former smokers (246/2,885, 8.5%)	2.34 (1.91, 2.87)	<0.001	1.19 (0.91, 1.55)	0.212
Current smokers (160/2,067, 7.7%)	2.11 (1.68, 2.63)	<0.001	1.46 (1.10, 1.96)	0.010

Values are presented as odds ratio (95% confidence interval).

1 Values in parentheses are presented as (number of participants/overall group size, %).

2 Covariates in the multivariable model include age, a continuous variable, as well as the categorical variables of sex, diabetes mellitus, hypertension, hyperlipidemia, obesity, family history of coronary artery disease, and C-reactive protein ≥2 mg/L.

**Table 4.** Coronary computed tomographic angiographic findings according to smoking status and amount

Characteristics	Smoking status/amount					p-value
	Never smokers (n=4,333)	Light former smokers (n=1,291)	Heavy former smokers (n=1,383)	Light current smokers (n=848)	Heavy current smokers (n=1,055)	
Coronary artery calcium score						
Mean±SD	24.6±118.9	45.6±179.6	70.8±229.0	37.0±181.9	57.8±162.8	<0.001
0	3,316 (76.9)	814 (63.3)	739 (53.7)	586 (69.4)	578 (55.1)	<0.001
1-10	314 (7.3)	132 (10.3)	149 (10.8)	80 (9.5)	108 (10.3)	
11-100	435 (10.1)	216 (16.8)	281 (20.4)	105 (12.4)	208 (19.8)	
101-400	181 (4.2)	91 (7.1)	140 (10.2)	59 (7.0)	118 (11.2)	
>400	66 (1.5)	33 (2.6)	67 (4.9)	14 (1.7)	37 (3.5)	
Any coronary plaque	1,067 (24.6)	490 (38.0)	664 (48.0)	274 (32.3)	497 (47.1)	<0.001
Plaque characteristics						
Calcified	976 (22.5)	464 (35.9)	629 (45.5)	251 (29.6)	454 (43.0)	<0.001
Non-calcified	163 (3.8)	64 (5.0)	131 (9.5)	42 (5.0)	106 (10.0)	<0.001
Mixed	88 (2.0)	36 (2.8)	89 (6.4)	31 (3.7)	81 (7.7)	<0.001
Obstructive coronary artery disease	166 (3.8)	76 (5.9)	159 (11.5)	48 (5.7)	103 (9.8)	<0.001

Values are presented as number (%). SD, standard deviation.

**Table 5.** Association of coronary computed tomographic angiographic findings with smoking status and amount

Variables^1^	Univariable	p-value	Multivariable^2^	p-value
Any coronary plaque				
Never smokers (1,067/4,333, 24.6%)	1.00 (reference)	-	1.00 (reference)	-
Light former smokers (490/1,291, 38.0%)	1.87 (1.64, 2.14)	<0.001	1.04 (0.87, 1.24)	0.672
Heavy former smokers (664/1,383, 48.0%)	2.83 (2.49, 3.21)	<0.001	1.16 (0.98, 1.38)	0.083
Light current smokers (274/848, 32.3%)	1.46 (1.25, 1.71)	<0.001	1.24 (1.02, 1.52)	0.033
Heavy current smokers (497/1,055, 47.1%)	2.73 (2.37, 3.13)	<0.001	1.57 (1.31, 1.89)	<0.001
Calcified plaque				
Never smokers (976/4,333, 22.5%)	1.00 (reference)	-	1.00 (reference)	-
Light former smokers (464/1,291, 35.9%)	1.93 (1.69, 2.21)	<0.001	1.05 (0.88, 1.25)	0.601
Heavy former smokers (629/1,383, 45.5%)	2.87 (2.53, 3.26)	<0.001	1.16 (0.98, 1.38)	0.092
Light current smokers (251/848, 29.6%)	1.45 (1.23, 1.70)	<0.001	1.21 (0.98, 1.48)	0.071
Heavy current smokers (454/1,055, 43.0%)	2.60 (2.26, 2.99)	<0.001	1.46 (1.22, 1.76)	<0.001
Non-calcified plaque				
Never smokers (163/4,333, 3.8%)	1.00 (reference)	-	1.00 (reference)	-
Light former smokers (64/1,291, 5.0%)	1.33 (0.99, 1.79)	0.056	0.95 (0.66, 1.35)	0.766
Heavy former smokers (131/1,383, 9.5%)	2.68 (2.11, 3.40)	0.001	1.57 (1.15, 2.14)	0.005
Light current smokers (42/848, 5.0%)	1.33 (0.94, 1.89)	0.105	1.21 (0.81, 1.81)	0.356
Heavy current smokers (106/1,055, 10.0%)	2.86 (2.22, 3.69)	<0.001	2.03 (1.47, 2.81)	<0.001
Mixed plaque				
Never smokers (88/4,333, 2.0%)	1.00 (reference)	-	1.00 (reference)	-
Light former smokers (36/1,291, 2.8%)	1.38 (0.93, 2.05)	0.105	0.77 (0.49, 1.21)	0.256
Heavy former smokers (89/1,383, 6.4%)	3.32 (2.46, 4.48)	<0.001	1.43 (0.98, 2.09)	0.064
Light current smokers (31/848, 3.7%)	1.83 (1.21, 2.78)	0.004	1.48 (0.92, 2.38)	0.110
Heavy current smokers (81/1,055, 7.7%)	4.01 (2.94, 5.47)	<0.001	2.17 (1.47, 3.20)	<0.001
Obstructive coronary artery disease				
Never smokers (166/4,333, 3.8%)	1.00 (reference)	-	1.00 (reference)	-
Light former smokers (76/1,291, 5.9%)	1.57 (1.19, 2.08)	0.002	0.90 (0.64, 1.26)	0.532
Heavy former smokers (159/1,383, 11.5%)	3.26 (2.60, 4.09)	<0.001	1.45 (1.08, 1.94)	0.013
Light current smokers (48/848, 5.7%)	1.51 (1.08, 2.10)	0.015	1.31 (0.89, 1.92)	0.168
Heavy current smokers (103/1,055, 9.8%)	2.72 (2.10, 3.51)	<0.001	1.60 (1.16, 2.20)	0.004

Values are presented as odds ratio (95% confidence interval).

1 Values in parentheses are presented as (number of participants/overall group size, %).

2 Covariates in the multivariable model include age, a continuous variable, as well as the categorical variables of sex, diabetes mellitus, hypertension, hyperlipidemia, obesity, family history of coronary artery disease, and C-reactive protein ≥2 mg/L.

## Supplementary materials

Supplementary Material 1. Study flow

Supplementary Material 2. Baseline characteristics of subjects with and without CCTA
