# Supplementary figures and images for: Association between smoking status and subclinical coronary atherosclerosis in asymptomatic Korean individuals
Source: Epidemiol Health. 2024 Jul 16;46:e2024064. doi: 10.4178/epih.e2024064 (PMC11576522; doi:10.4178/epih.e2024064)

**Supplementary Material 1. Study flow**


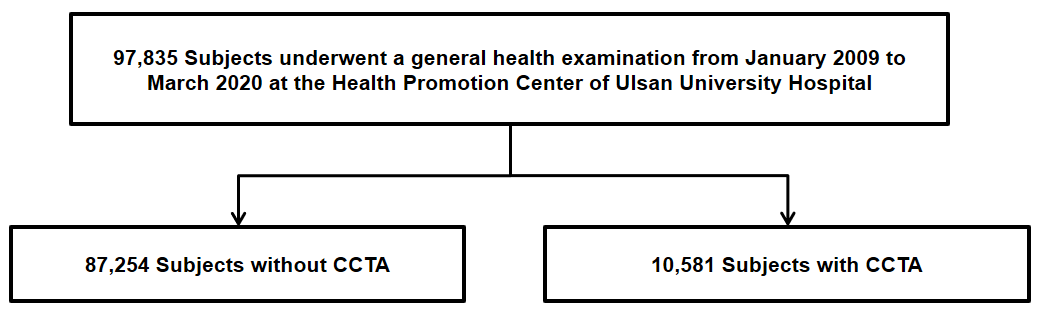


CCTA = coronary computed tomographic angiography

Supplement: Supplementary Material 1. — Study flow [file epih-46-e2024064-Supplementary-1.docx]
