# Supplementary material for: Association between smoking status and subclinical coronary atherosclerosis in asymptomatic Korean individuals
Source: Epidemiol Health. 2024 Jul 16;46:e2024064. doi: 10.4178/epih.e2024064 (PMC11576522; doi:10.4178/epih.e2024064)
Supplement: Supplementary Material 2. — Baseline characteristics of subjects with and without CCTA [file epih-46-e2024064-Supplementary-2.docx]

**Supplementary Material 2. Baseline characteristics of subjects with and without CCTA**

| Variables | Overall population (n=97,835) | No CCTA  (n=87,254) | CCTA (n=10,581) | P Value |
| --- | --- | --- | --- | --- |
| Demographics |  |  |  |  |
| Age, years | 47.9±9.6 | 47.3±9.5 | 52.5±8.5 | <0.001 |
| Men, no. (%) | 61,328 (62.7) | 54,358 (62.3) | 6,970 (65.9) | <0.001 |
| Clinical characteristics or coexisting conditions | | | |  |
| Body mass index, kg/m^2^ | 23.8±3.0 | 23.7±3.0 | 24.1±2.9 | <0.001 |
| Waist circumference, cm | 83.7±8.0 | 83.5±8.0 | 85.2±7.9 | <0.001 |
| Systolic blood pressure, mmHg | 121.8±13.6 | 121.5±13.5 | 123.9±14.0 | <0.001 |
| Diastolic blood pressure, mmHg | 77.2±9.3 | 77.1±9.3 | 78.3±9.4 | <0.001 |
| Diabetes mellitus, no. (%) | 8,145 (8.3) | 6,825 (7.8) | 1,320 (12.5) | <0.001 |
| Hypertension, no. (%) | 20,061 (20.5) | 16,728 (19.2) | 3,333 (31.5) | <0.001 |
| Hyperlipidemia, no. (%) | 13,599 (13.9) | 11,683 (13.4) | 1,916 (18.1) | <0.001 |
| Obesity^a^, no. (%) | 30,365 (31.0) | 26,753 (30.7) | 3,612 (34.1) | <0.001 |
| Smoking status, no. (%) |  |  |  | <0.001 |
| Current smoker | 23,096 (24.4) | 20,620 (24.4) | 2,476 (24.0) |  |
| Former smoker | 25,025 (26.4) | 21,854 (25.9) | 3,171 (30.8) |  |
| Never smoker | 46,537 (49.2) | 41,882 (49.6) | 4,655 (45.2) |  |
| Previous history of CAD, no. (%) | 1,296 (1.7) | 1,044 (1.5) | 252 (2.8) | <0.001 |
| Previous stroke, no. (%) | 303 (0.8) | 262 (0.8) | 41 (0.8) | 0.976 |
| Family history of CAD^b^, no. (%) | 4,584 (5.8) | 3,748 (5.4) | 836 (9.2) | <0.001 |
| Fasting blood glucose, mg/dL | 94.7±18.5 | 94.5±18.1 | 96.1±21.6 | <0.001 |
| Glycated hemoglobin (%) | 5.6±0.7 | 5.6±0.7 | 5.7±0.8 | <0.001 |
| Total cholesterol, mg/dL | 194.1±35.7 | 194.1±35.5 | 193.9±37.6 | 0.723 |
| LDL cholesterol, mg/dL | 54.8±14.8 | 55.1±14.8 | 52.8±14.6 | <0.001 |
| HDL cholesterol, mg/dL | 122.8±33.0 | 122.4±32.9 | 125.9±34.0 | <0.001 |
| Triglyceride, mg/dL | 115.6±76.9 | 115.3±76.8 | 118.1±77.8 | 0.001 |
| Creatinine, mg/dL | 0.9±0.3 | 0.9±0.3 | 0.9±0.2 | 0.280 |
| Ejection fraction, % | 64.3±4.9 | 64.2±4.9 | 64.3±4.9 | 0.366 |
| ASCVD risk score | 4.1±5.9 | 3.9±5.7 | 6.3±6.9 | <0.001 |

Values are given as mean±standard deviation or number (%).

^a^Obesity was defined as a body mass index ≥25 kg/m^2^

^b^Coronary artery disease in a first-degree relative of any age.

ASCVD = atherosclerotic cardiovascular disease; CAD = coronary artery disease; CCTA = coronary computed tomography angiography; HDL = high-density lipoprotein; LDL = low-density lipoprotein.
